# Metabolic dysfunction-associated liver disease and biliary lithiasis in children with Down Syndrome: a retrospective study

**DOI:** 10.1590/1984-0462/2025/43/2024130

**Published:** 2025-01-17

**Authors:** Renato Nisihara, Nayane Hiba Fuga, Liete Maia, Marcelo Gasparin Mansur, Fernanda Liberatti, Maiana Rossasi, Nanci Oliveira

**Affiliations:** a Universidade Positivo, Curitiba, PR, Brazil.; b Universidade Federal do Paraná, Curitiba, PR, Brazil.

**Keywords:** Down syndrome, Liver, Steatosis, Risk factors, Síndrome de Down, Fígado, Esteatose, Fatores de risco

## Abstract

**Objective:**

To investigate the presence of metabolic dysfunction-associated fatty liver disease (MAFLD) and gallbladder abnormalities in a sample of people with Down syndrome in Brazil.

**Methods:**

This is a retrospective study using medical charts involving Down syndrome patients, diagnosed by karyotype, aged over 5 years, who underwent abdominal ultrasound and were monitored by the same professional in a clinic in Curitiba, Brazil. Data spanned January 1995 to September 2023; all cases with no use of alcohol or hepatotoxic medications.

**Results:**

A total of 124 patients were evaluated, 64 (51.6%) being females. Ages varied between 5 and 30 years (average of 13.2±5.7 years). Body mass index ranged from 14.08 to 44.98 kg/m^2^, with 27 (21.7%) patients being overweight and 42 (33.8%) obese. The frequency of increased total cholesterol and triglyceride concentrations was significantly higher in children. No age or sex differences were seen in low- and high-density lipoprotein cholesterol. Alanine transferase (ALT) and aspartate transferase (AST) levels were within normal ranges and showed no differences concerning sex or age. Four patients (3.2%) had diagnosis of MAFLD. The prevalence of gallstone varied widely in terms of the number and size of stones among patients, affecting 11 (8.9%) of them, with no significant difference in age or sex.

**Conclusions:**

MAFLD was found in 3.2% of the individuals, while gallstone disease was identified in 8.9% of the cases studied. Moreover, we noted a significant presence of risk factors associated with MAFLD, with dyslipidemia being the predominant factor.

## RESUMO

**Objetivo:**

Investigar a presença de doença hepática gordurosa associada à disfunção metabólica (MAFLD) e anormalidades da vesícula biliar em uma amostra de pessoas com síndrome de Down no Brasil.

**Métodos:**

Trata-se de um estudo retrospectivo, utilizando prontuários médicos, envolvendo pacientes com síndrome de Down, diagnosticados por cariótipo, com idade superior a 5 anos, que realizaram ultrassonografia abdominal e foram acompanhados pelo mesmo profissional em um consultório em Curitiba, Brasil. Os dados abrangeram o período de janeiro de 1995 a setembro de 2023; todos os casos sem uso de álcool ou medicamentos hepatotóxicos.

**Resultados:**

Foram avaliados 124 pacientes, sendo 64 (51,6%) do sexo feminino. As idades variaram entre 5 e 30 anos (média de 13,2±5,7 anos). O índice de massa corporal variou de 14,08 a 44,98 kg/m^2^, sendo que 27 (21,7%) pacientes estavam com sobrepeso e 42 (33,8%) com obesidade. A frequência de concentrações aumentadas de colesterol total e triglicerídeos foi significativamente maior em crianças. Não foram observadas diferenças de idade ou sexo nas concentrações de colesterol de lipoproteína de baixa e alta densidades. Os níveis de aspartato transferase (AST) e alanina transferase (ALT) estavam normais e não apresentaram diferenças em relação ao sexo ou idade. Quatro pacientes (3,2%) tiveram diagnóstico de MAFLD. A prevalência de cálculo biliar variou amplamente em termos de número e tamanho dos cálculos entre os pacientes, afetando 11 (8,9%) deles, sem diferença significativa em idade ou sexo.

**Conclusões:**

MAFLD foi encontrada em 3,2% dos indivíduos, enquanto cálculo biliar foi identificado em 8,9% dos casos estudados. Além disso, notamos uma presença significativa de fatores de risco associados à MAFLD, com dislipidemia sendo o fator predominante.

## INTRODUCTION

Down syndrome (DS) stands as the most prevalent genetic contributor to cognitive impairment worldwide, impacting between 1 in 400 and 1 in 1500 individuals based on demographic factors, maternal age, and prenatal screening protocols.^1^ The primary associated anomalies are cardiac, followed by digestive, musculoskeletal, urinary, and respiratory system.^1^ Notably, congenital heart diseases are the most frequently observed. In adulthood, individuals with DS exhibit a higher propensity for overweight or obesity compared to the general population.^2^ Adolescents with DS were two to three times more likely to be obese than adolescents in the general population.^3^ People with DS can exhibit less favorable blood lipid levels than their counterparts, especially in terms of high-density lipoprotein cholesterol (HDL-C), triglycerides, and total cholesterol (TC), regardless of age grouping, which emphasize the need for vigilant monitoring of lipid profiles in this population.^4^

Metabolic dysfunction-associated liver disease (MAFLD), previously known as nonalcoholic fatty liver disease, is one of the most common hepatic diseases in individuals with overweight or obesity and there are evidence-based guidelines on the screening and follow-up of MASLD in individuals with overweight or obesity. Diagnosis of MAFLD requires the exclusion of other chronic liver conditions, including excessive alcohol consumption.^5^ The prevalence of MAFLD in children and adolescents ranges from 7.6% in general populations to 34.2% in obese populations.^6^ Those with MAFLD face heightened risks of various extrahepatic conditions, notably type 2 diabetes mellitus and cardiovascular disease, leading to increased overall mortality. A study that investigated MAFLD in DS children reported that independently of the obese phenotype, children with DS display a greater risk of developing MAFLD than the general pediatric population.^7^ The higher prevalence of overweight and obesity in individuals with DS is likely due to a combination of factors, including a lower resting metabolic rate, reduced physical activity, higher consumption of energy-dense and nutrient-poor foods, and hypothyroidism.^2-4^

Feeding difficulties and gastrointestinal issues are prevalent anomalies in individuals with DS and greatly affect their daily functioning. Researchers examined clinical and ultrasound data on 547 DS patients aged between one day and three years (average age: five months). In this study, they discovered gallbladder abnormalities in 9.1% of patients, with 6.9% showing gallstones (lithiasis) and 2.1% exhibiting biliary sludge.^8^ Herein, we assessed the presence of MAFLD and gallbladder abnormalities in a sample of people with DS in Brazil.

## METHOD

This is a retrospective study approved by the Institutional Research Ethics Committee under protocol 5.659.768. All participants signed informed consent.

The study included a convenience sample of patients diagnosed with DS by karyotype, aged over 5 years, who underwent an abdominal ultrasound examination (after the age of inclusion) and were monitored by the professional who provided the included data. All participants attended a single reference clinic in Curitiba, Brazil. The study was conducted using a survey of clinical charts from January 1995 to September 2023. None of the patients were alcoholics or used hepatotoxic medications.

Collected data consisted of epidemiological data (gender, age), height and weight for body mass index (BMI) calculation, and the presence of comorbidities. BMI was categorized based on the World Health Organization growth curves using a calculator (BVS, 2023).

Laboratory tests as levels of TC, HDL-C, low-density lipoprotein cholesterol (LDL-C), and triglycerides were collected from medical charts at the same time as the abdominal ultrasound was performed. The classification of lipid profile alterations followed the guidelines outlined in the Brazilian Guideline on Dyslipidemia and Prevention of Atherosclerosis.^9^

The abdominal ultrasound was conducted at a private clinic adhering to the guidelines set forth by the Brazilian College of Radiology and Imaging Diagnosis.^10^ Information concerning hepatic steatosis, gallstones, and other relevant details from the patients’ medical records was extracted in the ultrasound report. Identification of MAFLD was determined following the International Consensus guidelines.^11^ The diagnosis of MAFLD was established based on radiological evidence of fatty liver changes and categorized as mild, moderate, or severe. A histological examination of the liver biopsy was not performed as it is an invasive procedure.

According to the data distribution of the sample, the unpaired t-test and the Mann-Whitney U test were used to compare numerical data, and Spearman’s and Pearson’s tests were employed to examine correlations. The adopted significance was 5%. Tests were performed with GraphPad Prism software version 8.0.0 for Windows (GraphPad Software, San Diego, California USA, www.graphpad.com).

## RESULTS

In this study, 124 patients were evaluated, 64 (51.6%) females and 60 males. Ages varied between 5 and 30 years, with an average age of 13.2 years, standard deviation (±) 5.7. Among them, 34 (27.5%) were children (aged between 2–9 years), 72 (58.0%) were adolescents (10–19 years), and 18 (14.5%) were over 19 years old.

The most prevalent comorbidities discovered were hypothyroidism (25.8%), congenital heart disease (25.8%), attention deficit hyperactivity disorder (3.2%), and type 2 diabetes mellitus (1.6%). No significant association was observed between the presence of any comorbidity and findings related to MAFLD.

Table 1 ^12^ shows the data regarding BMI and laboratorial findings. In terms of BMI, patient values ranged from 14.08 to 44.98 kg/m^2^. Among them, 27 (21.7%) were classified as overweight, while 42 (33.8%) were classified as obese. A significant difference was observed when comparing the sexes, with women showing higher values of BMI. Among women, 39/64 (60.9%) were classified as obese or overweight, while men, 24/60 (40%) were classified above the adequate weight (p=0.020). These findings occurred in all ages as demonstrated in Table 1.

**Table 1:** Clinical and demographical profile of studied patients, according to age group. Data are expressed in numbers (%).

Characteristics*	Children (5–9 years) n=34	Adolescents (10–19 years) n=72	Adults (>19 years) n=18
Body mass index –kg/m^2^			
Obesity^†^	13 (38.2)	16 (22.2)	10 (55.5)
Overweight	8 (23.5)	17 (23.6)	1 (5.5)
Normal	12 (35.3)	36 (50.0)	7 (38.9)
Thinness	1 (2.9)	3 (4.2)	0 (0.0)
Total cholesterol^‡^			
Adequate	17 (50.0)	44 (61.1)	16 (88.9)
High	17 (50.0)	28 (38.9)	2 (11.1)
High density lipoprotein (HDL)			
Adequate	27 (79.4)	51 (70.8)	11 (61.6)
Low	7 (20.5)	21 (29.2)	7 (38.9)
Low density lipoprotein (LDL)			
Adequate	22 (64.7)	56 (77.8)	16 (88.9)
High	12 (35.3)	16 (22.2)	2 (11.1)
Total triglycerides^§^			
Adequate	14 (41.2)	41 (56.9)	17 (94.4)
High	20 (58.8)	31 (43.0)	1 (5.5)
Alanine transferase (ALT)			
Adequate	31 (91.2)	69 (95.8)	17 (94.4)
High	3 (8.8)	3 (4.2)	1 (5.5)
Aspartate transferase (AST)			
Adequate	27 (79.4)	70 (97.2)	18 (100.0)
High	7 (20.6)	2 (2.8)	0 (0.0)

*The laboratorial reference values used were adjusted according to age^12^;

^†^Adults present more obesity compared to other groups (p=0.038);

^‡^Children show elevate total cholesterol (p=0.016) comparing the age groups;

^§^Children show elevate triglycerides (p<0,001) comparing the age groups.

When stratifying TC according to references adjusted for age, 47 (37.9%) cases showed values above the references. This was significantly observed in children, of whom 50.0% had high levels of TC compared to adolescents or adults (p=0.016); there were no significant differences between the sexes. In our sample, 30 (24.2%) cases showed high levels of LDL-C and 35 (28.2%) had HDL-C below the reference values. No significant difference was observed between LDL-C or HDL-C values when comparing the ages and sexes of the individuals studied.

Regarding triglyceride levels, 52 (41.9%) patients were above reference values. Additionally, we found that 58.8% of the children had triglycerides above reference values, significantly more than adolescents and adults (p=0.00022), also without significant alterations between the sexes.

As for hepatic enzymes, aspartate transferase (AST) ranged from 10.4–79.0 UI/mL and alanine transferase (ALT) ranged from 8.2–84.7 UI/mL. Among them, 9 (7.3%) had AST and 7 (5.7%) had ALT above the reference values adjusted for age. There was no difference between sexes or age.

Based on the abdominal ultrasound findings, hepatic steatosis was observed in 4 (3.2%) patients diagnosed with MAFLD. It is evident that they were older than the rest of the analyzed group. Additionally, they presented with other conditions such as increase of BMI, hypothyroidism, and altered lipid profile. Detailed data regarding these patients can be found in Table 2.

**Table 2:** Data from the four patients diagnosed with metabolic dysfunction-associated fatty liver disease (MAFLD).

Gender	Age at diagnosis	BMI (kg/m^2^)	TC (mg/dL)	HDL (mg/dL)	LDL (mg/dL)	Triglycerides (mg/dL)	ALT/AST (UI/mL)	Ultrasound	Comorbidities
Male	12 years	32.4	188	27	112.4	243	18/31	Mild steatosis without gallstones	Obstructive sleep apnea syndrome
Female	12 years	44.9	153	50	84.2	94	30/68	Moderate steatosis without gallstones	None
Female	15 years	27.8	254	74	98.6	121	18/22	Mild steatosis without gallstones	Hypothyroidism
Female	21 years	32.5	173	63	86.8	116	12/15	Mild steatosis with gallstones	Hypothyroidism

BMI: body mass index; TC: total cholesterol; HDL: high-density lipoprotein; LDL: low-density lipoprotein; ALT: alanine transferase; AST: aspartate transferase.

The prevalence of gallstone disease varied widely in terms of the number and size of stones across patients, affecting 11 (8.9%) of them, with no significant difference in age (p=0.920). Among these cases, there were 8 female and 3 male patients with gallstone disease (p=0.340).

## DISCUSSION

MAFLD is becoming more significant in young individuals’ health, closely linked to their lifestyles. Factors such as obesity, unbalanced diet, and a sedentary lifestyle play crucial roles. MAFLD can advance to severe liver damage and cirrhosis, underscoring the critical need for early prevention and the promotion of healthy habits starting from childhood. This is primarily pertinent for individuals with DS, as these recommendations are likely to be even more vital, particularly concerning the management of increased body weight.

This study stands out due to the notable prevalence of DS individuals with overweight and obesity in all age groups. Taking into account only children, it was observed that 23.5% of the DS children studied were overweight and 38.2% were obese. In contrast, data from the general pediatric population in the same region showed lower rates, with 9.0% classified as overweight and 5.9% as obese.^13^ The rise in obesity rates in both developed and developing countries across all age groups is attributed to sedentary behaviors and the consumption of high energy density foods.^13^ Children and adolescents with DS seem to experience elevated rates of overweight and obesity compared to their peers in the general population.^2^ Potential contributors to obesity in this group include elevated leptin levels, reduced resting energy expenditure, decreased physical activity levels, unhealthy dietary habits, and associated medical conditions. Obesity in young individuals with DS is associated with an increased likelihood of developing dyslipidemia, hyperinsulinemia, obstructive sleep apnea, and gait abnormalities.^2^ In addition, a significant increase in obesity among DS young people was observed compared to other age groups. A recent meta-analysis concluded that exercise, physical activity, and sport have a positive and significant effect on fitness in adults with DS, specifically on strength, balance, body composition, cardiorespiratory fitness, flexibility, and functional capacity, improving their quality of life.^14^ Certainly, the early introduction of a routine of physical activities for DS children and adolescents should also produce physical progress and improvements in the quality of life of future adults.

Another notable finding in our study was the higher prevalence of overweight and obesity among women across all age groups. This may be linked to lower levels of physical activity or potentially higher leptin levels typically found in females.^15^ However, the literature still lacks sufficient studies to fully explain this observation.

In our study, dyslipidemia was found mainly in children. Dyslipidemia prevalence observed in our cases surpassed findings from a Mexican study,^16^ in which 6.5% of cases had high TC. A recent meta-analysis^17^ reported the findings in 15 studies on TC, HDL-C, LDL-C, and triglycerides, comparing DS with controls. Notably, the DS group exhibited less favorable lipid profiles compared to those of the control group, except for TC levels, although lower and seemingly favorable in the DS group. However, individuals with DS exhibited low levels of HDL-C and elevated triglyceride concentrations. It should be noted that this study did not perform analyses regarding the age of the cases studied and the great heterogeneity between the studies evaluated.^17^

On the other hand, the underlying reasons behind the tendency of DS individuals to have unfavorable lipid profiles remain unclear. It is possible that this is related to the higher prevalence of overweight and obesity, along with dietary and lifestyle habits. However, there is still some debate about whether higher BMI or nutritional status should be considered risk factors for dyslipidemia in DS.^17^

Our study included only patients who underwent an abdominal ultrasound, which was not standard for all cases attended in the clinic. Among those examined, 3.2% had hepatic steatosis. A cross-sectional study in Italy with 33 DS children (aged 5–17 years) found associations between overweight/obesity and hypertransaminasemia/dyslipidemia, suggesting routine monitoring of lipid and hepatic indicators, particularly in overweight/obese patients, for early detection of cardiovascular and non-alcoholic steatohepatitis risks.^18^ In Italy, another study involving 84 DS patients (aged 5–18 years) found that 64.3% of children with DS had MAFLD, regardless of whether they were obese.^7^ Differences in culture, lifestyle habits, and methodological approaches can contribute to these varying results.

Gallstone disease was detected in 8.9% of the cases analyzed, a figure consistent with findings from studies involving children with DS conducted by other researchers.^8^ The primary cause of biliary lithiasis, as observed in our study, was cholesterol stones, driven by elevated TC levels; 47 out of 124 patients (37.9%) had TC levels exceeding the age-adjusted reference values. Notably, of the 11 patients with gallstone disease, five had TC levels above these age-adjusted reference values.

The present study was limited by its retrospective approach within a single center. Additionally, we were unable to retrieve lifestyle data, such as dietary habits and exercise patterns, from the medical records. MAFLD diagnosis relied solely on ultrasound imaging; once a liver biopsy is invasive, it was not conducted. Notably, the strength of the study lies in its substantial sample size and comprehensive access to clinical and laboratory information concerning the patients under investigation.

Our findings underscore the critical need for healthcare providers to manage patients with DS, regardless of age, but mainly in children. It is necessary to remain vigilant about dyslipidemia and to order abdominal ultrasound assessments periodically. Moreover, fostering healthy habits and promoting early behavioral modifications among individuals with DS is paramount, supported by a collaborative, multidisciplinary team.

Concluding, in this sample of individuals with DS in Brazil, we observed a high prevalence of risk factors for MAFLD, primarily dyslipidemia, mainly in children. In addition, MAFLD was detected in 3.2% and gallstone disease was detected in 8.9% of the cases analyzed.

## Data Availability

The database that originated the article is available with the corresponding author. CAAE: 60266022.3.0000.0093
